# Supplementary figures and images for: Anti-asthmatic effect of nitric oxide metallo-donor FOR811A [cis-[Ru(bpy)2(2-MIM)(NO)](PF6)3] in the respiratory mechanics of Swiss mice
Source: PLoS One. 2021 Mar 12;16(3):e0248394. doi: 10.1371/journal.pone.0248394 (PMC7954307; doi:10.1371/journal.pone.0248394)

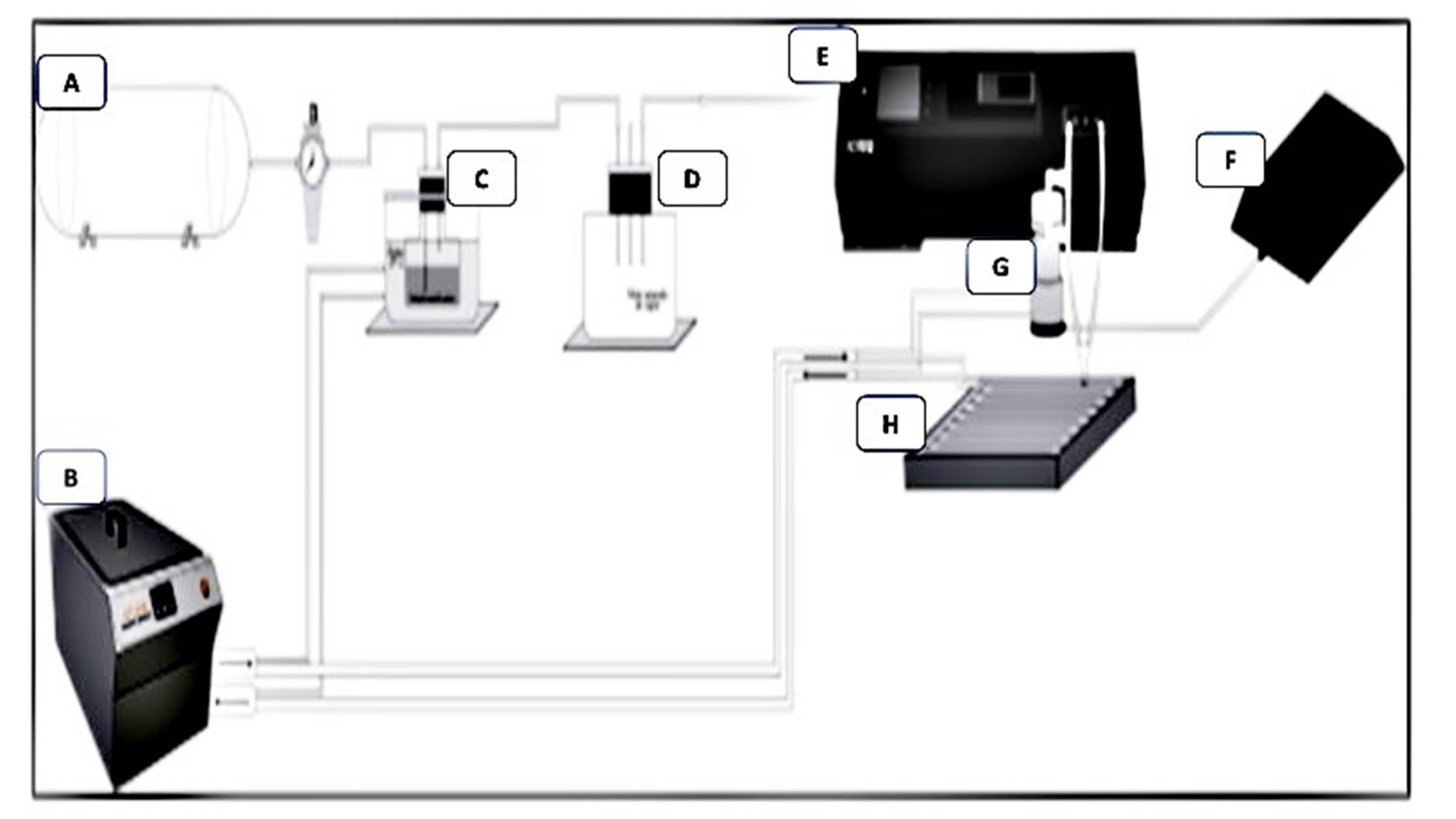

Supplement: S1 Fig — Through a carbogen cylinder (A), oxygenation was maintained at a ratio of 95%:5% (O2:CO2). An air purification unit (B) was attached to the equipment responsible for maintaining the heating and humidification of the air (C) and the depressurizer (D), mechanical fan for small animals (E) and ultrasonic nebulizer (F). Also, a reservoir containing bronchoconstrictor (G) and a bed with heating support (H) for maintaining the body at a temperature of 37°C were available. (TIF) [file pone.0248394.s001.tif]
